# Integrating fractional amplitude of low-frequency fluctuation and functional connectivity to investigate the mechanism and prognosis of severe traumatic brain injury

**DOI:** 10.3389/fneur.2023.1266167

**Published:** 2023-12-08

**Authors:** Biao Li, Wu-gen Li, Yao Guo, Yang Wang, Lu-yang Xu, Yuan Yang, Shi-guo Xu, Zi-long Tan, Yu-ran Mei, Kai-yang Wang

**Affiliations:** ^1^Department of Emergency, The Second Affiliated Hospital of Nanchang University, Nanchang, Jiangxi, China; ^2^Department of Emergency, Nanchang Hongdu Hospital of Traditional Chinese Medicine, Nanchang, Jiangxi, China; ^3^Department of Imaging, The Second Affiliated Hospital of Nanchang University, Nanchang, Jiangxi, China; ^4^Department of Neurosurgery, Shanghai Ninth People’s Hospital, Shanghai Jiao Tong University School of Medicine, Shanghai, China; ^5^Department of Neurosurgery, The Second Affiliated Hospital of Nanchang University, Nanchang, Jiangxi, China

**Keywords:** severe traumatic brain injury, functional magnetic resonance imaging, prognosis, fractional amplitude of low-frequency fluctuation, functional connectivity

## Abstract

**Objective:**

Functional magnetic resonance imaging (fMRI) has been used for evaluating residual brain function and predicting the prognosis of patients with severe traumatic brain injury (sTBI). This study aimed to integrate the fractional amplitude of low-frequency fluctuation (fALFF) and functional connectivity (FC) to investigate the mechanism and prognosis of patients with sTBI.

**Methods:**

Sixty-five patients with sTBI were included and underwent fMRI scanning within 14 days after brain injury. The patient’s outcome was assessed using the Glasgow Outcome Scale—Extended (GOSE) at 6 months post-injury. Of the 63 patients who met fMRI data analysis standards, the prognosis of 18 patients was good (GOSE scores ≥ 5), and the prognosis of 45 patients was poor (GOSE scores ≤  4). First, we apply fALFF to identify residual brain functional differences in patients who present different prognoses and conjoined it in regions of interest (ROI)-based FC analysis to investigate the residual brain function of sTBI at the acute phase of sTBI. Then, the area under the curve (AUC) was used to evaluate the predictive ability of the brain regions with the difference of fALFF and FC values.

**Results:**

Patients who present good outcomes at 6 months post-injury have increased fALFF values in the Brodmann area (7, 18, 31, 13, 39 40, 42, 19, 23) and decreased FC values in the Brodmann area (28, 34, 35, 36, 20, 28, 34, 35, 36, 38, 1, 2, 3, 4, 6, 13, 40, 41, 43, 44, 20, 28 35, 36, 38) at the acute phase of sTBI. The parameters of these alterations can be used for predicting the long-term outcomes of patients with sTBI, of which the fALFF increase in the temporal lobe, occipital lobe, precuneus, and middle temporal gyrus showed the highest predictive ability (AUC = 0.883).

**Conclusion:**

We provide a compensatory mechanism that several regions of the brain can be spontaneously activated at the acute phase of sTBI in those who present with a good prognosis in the 6-month follow-up, that is, a destructive mode that increases its fALFF in the local regions and weakens its FC to the whole brain. These findings provide a theoretical basis for developing early intervention targets for sTBI patients.

## Introduction

It has been reported that more than 50 million people suffer from traumatic brain injury (TBI) each year, of which severe TBI (sTBI) is a significant contributor to high mortality and morbidity worldwide (1). To the best of our knowledge, patients with sTBI are heterogeneous and encompass a cascade of brain disorders and secondary injuries (2, 3). With the goal of continuously optimizing the conditions for brain recovery, active brain function monitoring can provide a reliable basis for mechanism-guided management of sTBI (4).

With the advances in intensive care, especially neurocritical management, there is great potential for objectively evaluating the pathophysiology of patients with sTBI (5). However, in most developing countries, people have always been concerned that the treatment and cure come at the cost of an increase in the economic burden and the survivors with severe disabilities. Confounded by uncertainty in sTBI prognoses, many helpless trauma centers and family members have made decisions to withdraw from life-sustaining therapies. It has been reported that early withdrawal of life-sustaining therapies does not have worse outcomes than those making later decisions (6). In a recent study that included 41 randomized trials, the total mortality rate of sTBI was 23%, of which 63% was related to the withdrawal of life-sustaining therapies (7). Therefore, there is an unmet clinical need to identify and establish accurate sTBI predictive biomarkers that correlate with the sTBI pathologies at the acute phase (8).

Clinicians gradually realize that spontaneous brain activity in a resting state is distributed spatially and temporally in multiple brain regions of patients with sTBI (9, 10). With the help of functional magnetic resonance imaging (fMRI), people can identify and describe the widespread disruption of brain network mechanisms, such as the changes in functional connectivity and abnormal brain activation in patients with sTBI (11–13). However, most of the assessments were carried out in the rehabilitation stages of sTBI, and the timeliness of assessment is lost in the acute phase of sTBI. In recent days, studies have reported that there was a compensatory mechanism that several brain regions can converge into abnormal functional hubs in the acute phase of patients with sTBI, which provides a potential approach to predict the long-term outcome of patients (14). However, it is still not clear about the abnormal brain function patterns in which local brain regions play an important role in the formation of this compensation mechanism.

In the study of fMRI, a fractional amplitude of low-frequency fluctuation (fALFF) approach was proposed to reveal the spontaneous activity of the brain regionally (15). In addition, regions of interest (ROI)-based functional connectivity (FC) can be calculated to discrete the various brain functional states of the whole brain spatially (16). It has recently been found that sTBI patients who present a better prognosis showed a significant increase in regional and spatial values of the brain cortical network (11). Based on this further, we hypothesized that (1) changes in residual brain spontaneous activity after sTBI will disrupt the functional pattern of the whole brain, and (2) this early compensatory mechanism can be used for the long-term prognostic prediction of sTBI.

## Methods

### Participants

The patients with sTBI from January 2020 to July 2022 were enrolled and managed according to the 4th Brain Trauma Foundation Guidelines in the local neurotrauma center. To minimize the influence of structural distortion in functional analysis, we specifically included patients who present with closed brain injury and did not receive craniotomy. All sTBI patients were diagnosed with diffuse axonal injury and to restrain the abnormal blood oxygen signal noise caused by intracerebral hematoma or contusion lesions.

The eligibility criteria were as follows: (1) age 18–60 years old, with no history of brain injury; (2) meet the diagnostic criteria of sTBI at admission (GCS scores between 3 and 8 points); (3) fMRI scanning can be safely completed within 14 days post-injury, judged by doctors with a senior professional title. The exclusion criteria were as follows: (1) death before fMRI scanning; (2) cerebral hernia (bilateral dilated or fixed pupils), respiratory failure (require assisted ventilation) during hospitalization, or circulatory failure (unresponsive to vasopressor agents); (3) obvious hematoma or brain contusion lesions; (4) pregnant or lactating. Specifically, patients in the comatose stage were defined as having a GCS lower than 8 points, and we used command-following as a primary outcome measure for early emergence from coma in patients with acute severe TBI (17). This study obtained the informed consent of the patient's family members and the approval of the hospital ethics committee.

### Resting-state fMRI scanning

All patients with severe TBI underwent fMRI scanning as soon as they were stable to transport to the MRI scanner (at the acute phase of sTBI; within 14 days post-injury), as determined by local clinicians. We stop analgesics or sedatives the night before the scan. Resting-state fMRI (rs-fMRI) scan was performed by using a 3.0 Tesla system (Siemens, Germany) equipped with the gradient echo-planar parameters: (percent phase field view = 100, flip angle = 90°, TE = 30 ms, TR = 2,000 ms, FOV = 300 × 300 mm; matrix = 64 × 64, thickness = 3.5 mm, and slices = 33, 8 min) at the acute sTBI. During the scanning, all participants were lay supine with the head firmly fixed by a coil to minimize head motion. A compatible monitor is used to monitor the vital signs of all participants. We stop scanning for patients with abnormal vital signs (decreased pulse oxygen, abnormal blood pressure, etc.).

### Outcome assessment and grouping

All sTBI patients were treated according to the current institutional strategy and managed by a team of neurosurgeons with senior professional positions and full-time doctor titles. The Glasgow Outcome Scale Extended (GOS-E) scale was used to evaluate the functional outcome through outpatient service, WeChat, or telephone consultation at 6 months post-injury (where “8” = fully recovered and “1” = died). Accordingly, the prognosis was dichotomized into poor outcomes (GOSE 1–4) or good outcomes (GOSE 5–8). In this study, we divided sTBI patients into a “good outcome” group and a “bad outcome” group based on the outcomes obtained in 6-month follow-up.

### fMRI data pre-processing

All resting state fMRI data were preprocessed by Statistical Parametric Mapping 12 (SPM 12)^1^ performed on MATLAB 2016a (MathWorks, Natick, MA, United States). First, the first ten time points of the BOLD time series were discarded to eliminate the instability. The DPABI toolbox^2^ was used for data pre-processing, which included slice timing, head-motion correction, skull masking, and spatial normalization. To reduce the impact of head movements in the acute phase of sTBI, we used a higher-order framework (Friston 24 head motion parameters model) to regress out head motion effects and ruled out the participants who present with more than two degrees of rotation and 2 mm of translation (x, y, z). Then, we performed linear regression to remove spurious covariates along with temporal derivatives (cerebrospinal fluid signals). After that, these fMRI data were normalized to the Montreal Neurological Institute (MNI) template spatially re-sampled at a resolution of 3 × 3 × 3 mm^3^ space. In addition, the low-frequency drift and the high-frequency physiological noise have been reduced through bandpass filtration (0.01–0.1 Hz) and linearly detrended. Finally, the fMRI data was smoothed using a Gaussian kernel of 4 × 4 × 4 mm^3^ full width at half-maximum (FWHM).

### fALLF and FC analysis

At first, the 0.01–0.08 Hz frequency band power spectrum was retained as the amplitude of low-frequency fluctuations (ALFF). Then, we calculated a ratio of each participant’s ALFF to that of their entire 0.01–0.1 Hz frequency range as the fALFF value. After that, the spontaneous brain activity of the sTBI patients who presented with different prognoses was evaluated by fALFF. Brain regions with significant between-group differences of fALFF values were saved as seed points, and a 2.5 mm radius ROI region was set to calculate its FC to the whole brain. Finally, the difference connectome map of sTBI patients who present different prognoses was obtained. Pearson correlation coefficients between ROI and voxel were subjected to Fisher’s Z transformation to make the data fit a normal distribution.

### Statistical analysis

Continuous variables are expressed as mean SD and categorical variables as percentages. Normally distributed variables were compared using the one-way ANOVA test or Student’s *t*-test. Two-sample *t*-tests were used to evaluate the differences of voxel-wise in brain regions of each group. Multiple comparison correction was performed using the AlphaSim to correct FC values (for *p* < 0.05, voxels > 154). GraphPad Prism 9.0 software^3^ was conducted for data analysis. A *P*-value of < 0.05 was considered statistically significant.

We further constructed receiver operating characteristic (ROC) curves to determine the predictive value of the defined ROI, the differential FC, and the integration of fALFF and FC. The area under the ROC curve (AUC) (where 1.0 implies perfectible and 0.5 implies ineffective) and the sensitivity and specificity of different cutoff levels were calculated to evaluate the discriminative ability and predictive value.

## Results

### Participant

A total of 65 patients completed fMRI scans, and 63 sTBI patients (55 in a comatose state) entered the final analysis after the head movement data had been excluded. The demographic characteristics and the outcomes of the included participants are summarized in Table 1. There were no significant differences in gender, age, and admission time between sTBI patients who presented with different prognoses. However, there were statistically significant differences between the two groups (poor and good outcomes) in terms of discharge GOS-E (*P* < 0.001).

**Table 1:** Baseline characteristics of patients with sTBI.

Terms	Good outcomes (*n* = 18)	Poor outcomes (*n* = 45)	*P-*value
Age	50.24 ± 6.36	52.82 ± 12.53	0.29
Gender, male (%)	11 (61.11)	28 (62.22)	0.94
Admission GCS	6.27 ± 2.52	4.81 ± 2.48	0.04
Admission time, h	5.28 ± 1.24	6.17 ± 2.36	0.06
fMRI acquisition time, d	14.56 ± 5.47	15.82 ± 7.24	0.51
Discharge GOS-E	5.61 ± 1.87	2.45 ± 1.26	<0.001

sTBI, severe traumatic brain injury; GCS, Glasgow coma score; h, hours; fMRI, functional magnetic resonance imaging; d, days; GOS-E, extended Glasgow outcome score.

### Spontaneous brain activity alterations between the poor and good outcomes

**Figure 1:**
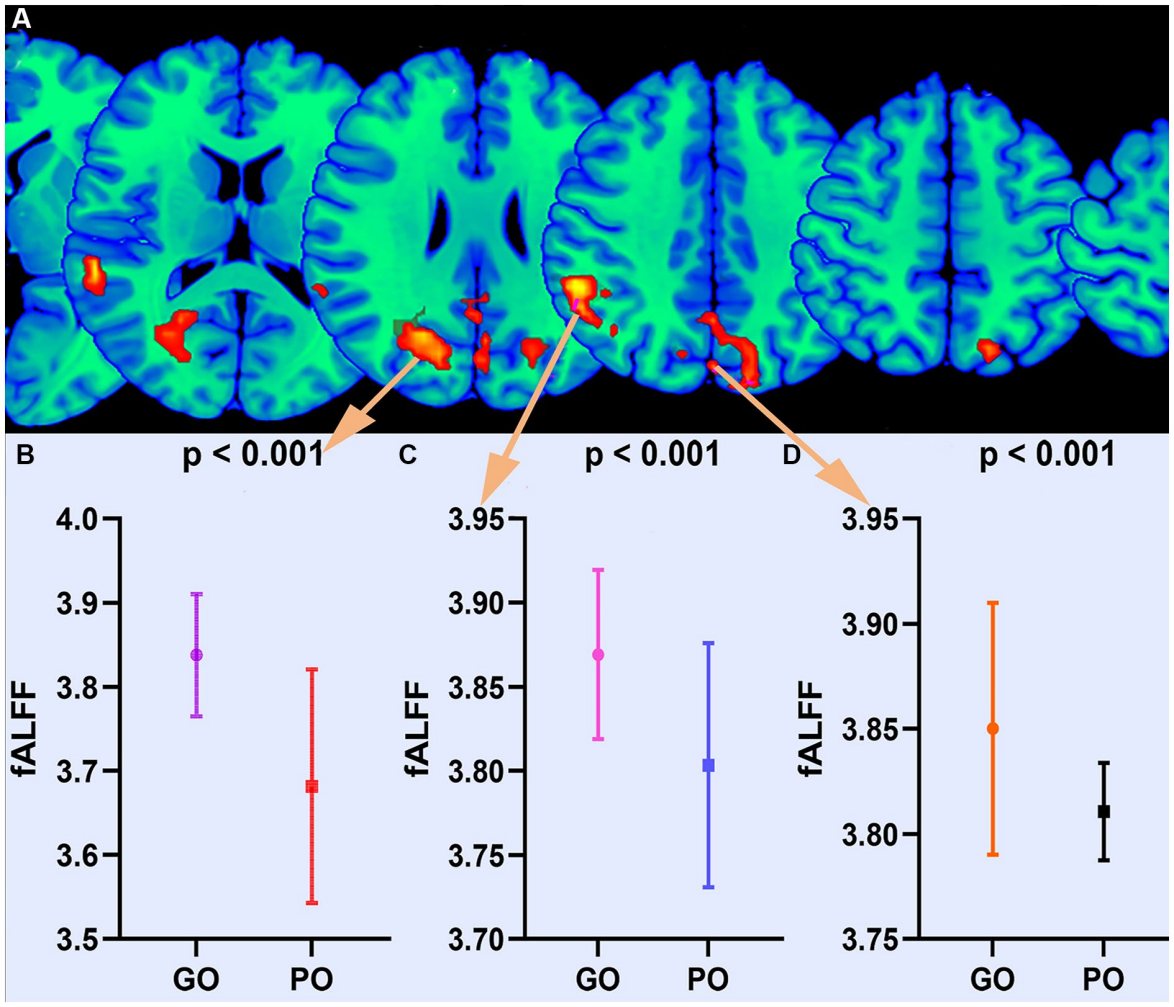
Altered fALFF between good outcome and poor outcome groups. **(A)** Differential brain regions of fALFF. **(B)** Statistical quantification of fALFF. GO, good outcome; PO, poor outcome.

The findings of the two-sample t-test in fALFF of the sTBI patients who present with good and poor outcomes are depicted in Figure 1 and Table 2 (upper). The spontaneous brain activity was significantly increased in sTBI patients who present with the good outcome than those present with the poor outcome in the temporal lobe, the occipital lobe, the precuneus, the middle temporal gyrus (peak point 21/−63/24, MNI coordinates); the gyrus, the superior temporal gyrus, the angular gyrus, the insula (peak point 57/−39/18, MNI coordinates); and the limbic lobe, the superior parietal gyrus, the cingulate gyrus, the posterior cingulate gyrus (peak point 0/−78/36, MNI coordinates) (AlphaSim corrected, *P* < 0.05). All voxels, MNI coordinate, and region information are available in Supplementary material.

**Table 2:** Altered fractional amplitude of low-frequency fluctuation and functional connectivity between good outcome and poor outcome groups.

Terms	Voxels	Areas	Peak MNI (X/Y/Z)	T	Alphasim *P*
fALFF: good outcome group > poor outcome group					
1	180	Temporal lobe, occipital lobe, precuneus, middle temporal gyrus, Brodmann Area (7, 18, 31)	21/−63/24	6.39	<0.05
2	165	Parietal lobe, inferior parietal lobule, supramarginal gyrus, superior temporal gyrus, angular gyrus, insula, Brodmann area (13, 39 40, 42)	57/−39/18	5.97	<0.05
3	223	Limbic lobe, superior parietal gyrus, cingulate gyrus, posterior cingulate gyrus, Brodmann area (19, 23)	0/−78/36	5.85	<0.05
Seed point FC: good outcome group < poor outcome group					
Seed point (21/−63/24)					
1	271	Pons, limbic lobe, uncus, parahippocampa, Brodmann area (28, 34, 35, 36)	−12/−18/−48	−5.49	<0.05
Seed point (21/−63/24)					
1	171	Limbic lobe, uncus, parahippocampa, brainstem, superior and inferior temporal gyrus, amygdala, Brodmann area (20, 28, 34, 35, 36, 38)	18/−12/−45	−3.60	<0.05
2	269	Parietal lobe, frontal lobe, rolandic operculum, precentral gyrus, postcentral gyrus, insula, supramarginal gyrus, inferior parietal lobule, transverse temporal gyrus, inferior frontal gyrus, Brodmann area (1, 2, 3, 4, 6, 13, 40, 41, 43, 44)	48/−3/9	−3.03	<0.05
Seed point (0/−78/36)					
1	343	Pons, limbic lobe, uncus, brainstem, fusiform gyrus, inferior temporal gyrus, parahippocampa, cerebellar tonsil, cerebellum posterior lobe, Brodmann area (20, 28 35, 36, 38)	−9/−18/−48	−4.57	<0.05

MNI, Montreal neurological institute; fALFF, fractional amplitude of low frequency fluctuation; FC, functional connectivity.

### The impact of altered spontaneous brain activity on the whole brain

**Figure 2:**
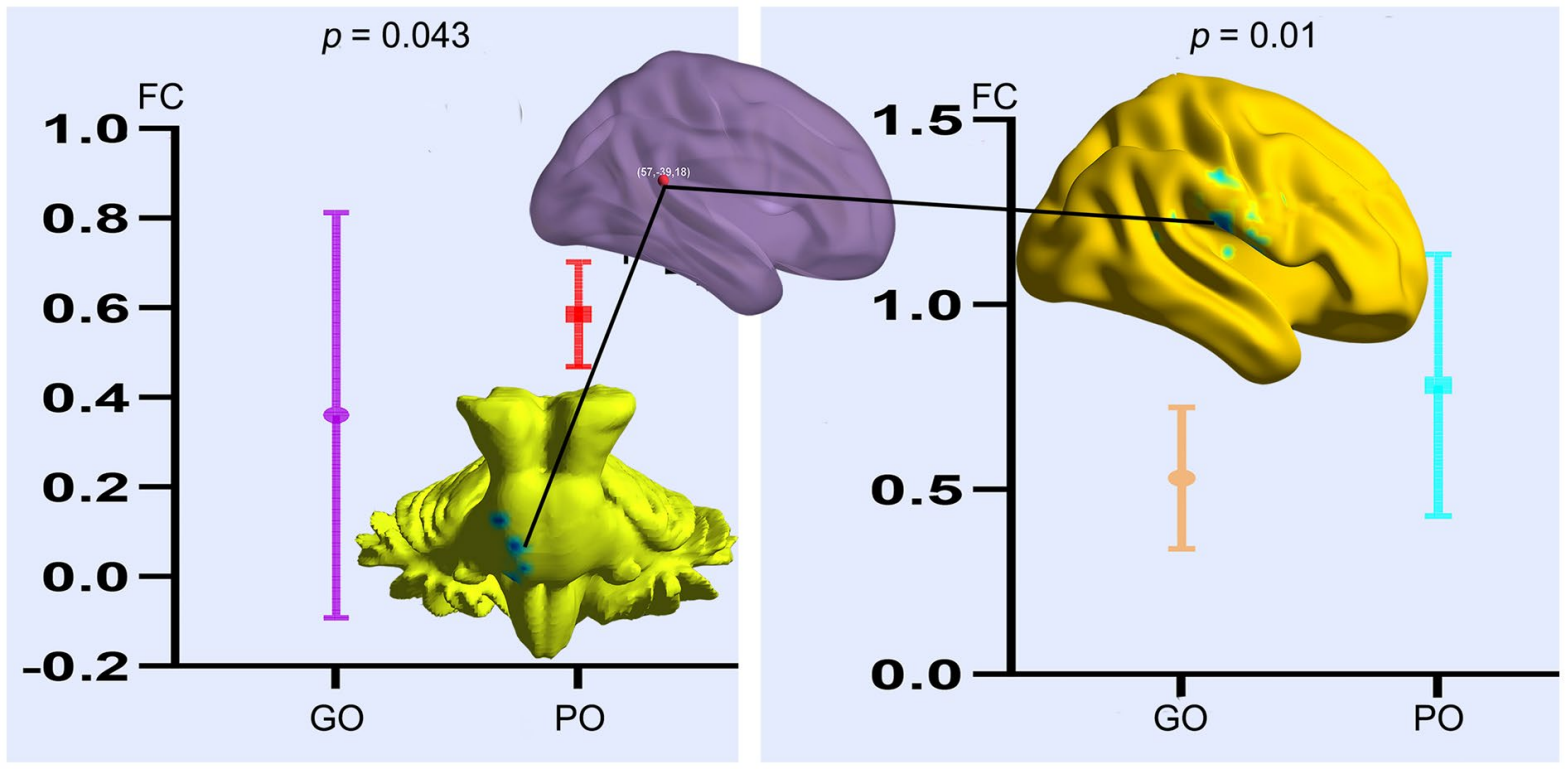
FC differences of altered spontaneous brain activity (ROI-2) between sTBI patients who present with different outcomes. GO, good outcome; PO, poor outcome.

**Figure 3:**
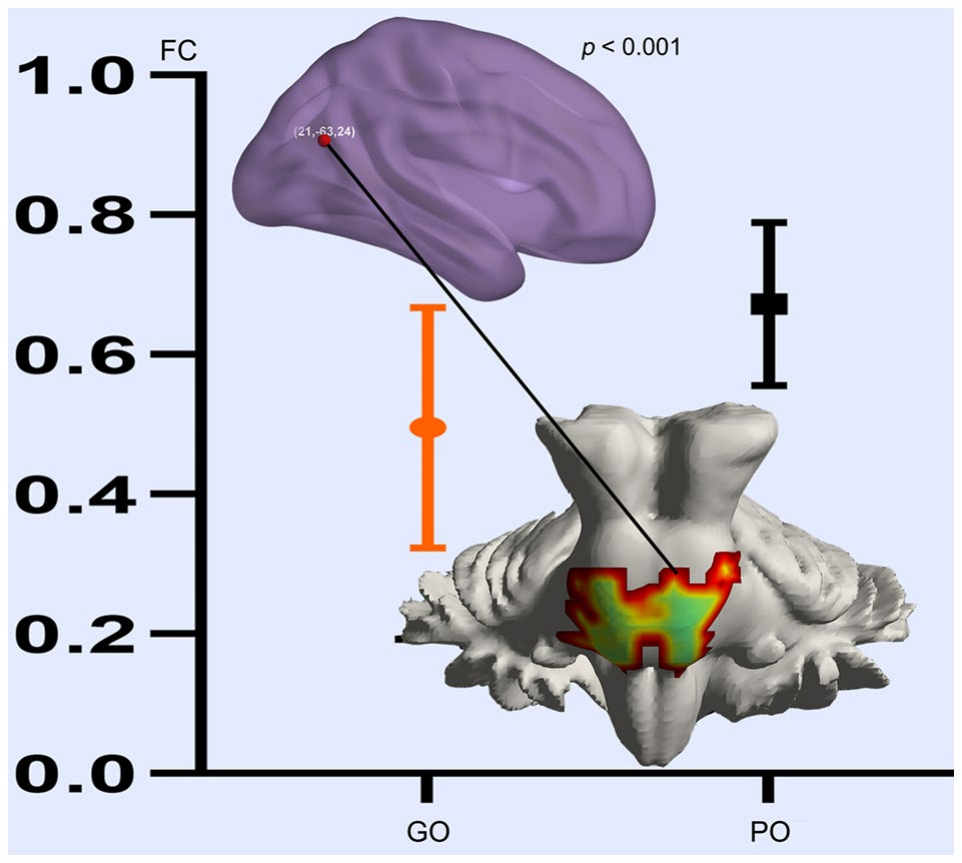
FC differences of altered spontaneous brain activity (ROI-1) between sTBI patients who present with different outcomes. GO, good outcome; PO, poor outcome.

**Figure 4:**
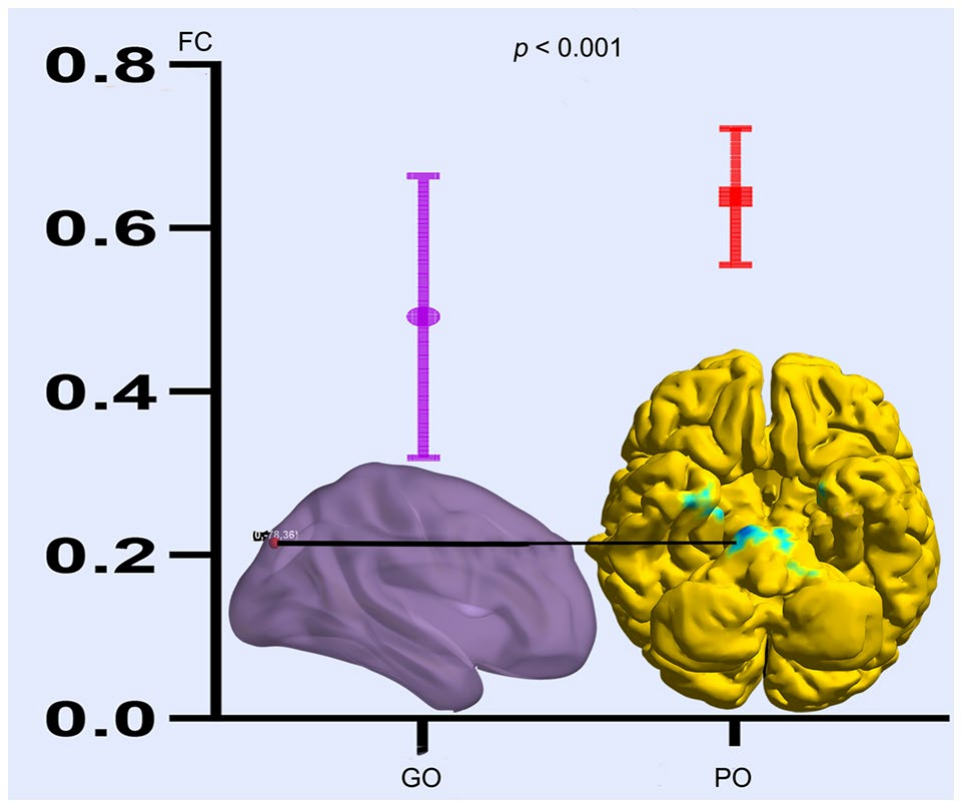
FC differences of altered spontaneous brain activity (ROI-3) between sTBI patients who present with different outcomes. GO, good outcome; PO, poor outcome.

Based on the altered fALFF value areas in Figures 1, 2, ROI (thereafter named ROI-1; ROI-2; ROI-3) located in posterior cortex brain areas (21/−63/24, MNI coordinates), (57/−39/18, MNI coordinates), and (0/−78/36, MNI coordinates) were extracted. The *Z*-value was used to quantify the differences in whole brain FC between sTBI patients who present different spontaneous brain activity (Table 2, lower). Compared to sTBI patients who present with the poor outcome, the FC in patients who present the good outcome were statistically decreased between the pons, the limbic lobe, the uncus, the parahippocampa, and the ROI-1 (Figure 3) (*P* < 0.05); compared to sTBI patients who present with the poor outcome, the FC in patient who present with the good outcome were statistically decreased between the limbic lobe, the uncus, the parahippocampa, the brainstem, the superior and inferior temporal gyrus, the amygdala, and the ROI-2, and statistically decreased between the parietal lobe, the frontal lobe, the rolandic operculum, the precentral gyrus, the postcentral gyrus, the insula, the supramarginal gyrus, the inferior parietal lobe, the transverse temporal gyrus, the inferior frontal gyrus, and the ROI-2 (Figure 2) (*P* < 0.05); compared to sTBI patients who present with the poor outcome, the FC in patient who present with the good outcome were statistically decreased between the pons, the limbic lobe, the uncus, the brainstem, the fusiform gyrus, the inferior temporal gyrus, the parahippocampa, the cerebellar tonsil, the cerebellum posterior lobe and the ROI-3 (Figure 4) (*P* < 0.05).

### Outcome prediction

**Figure 5:**
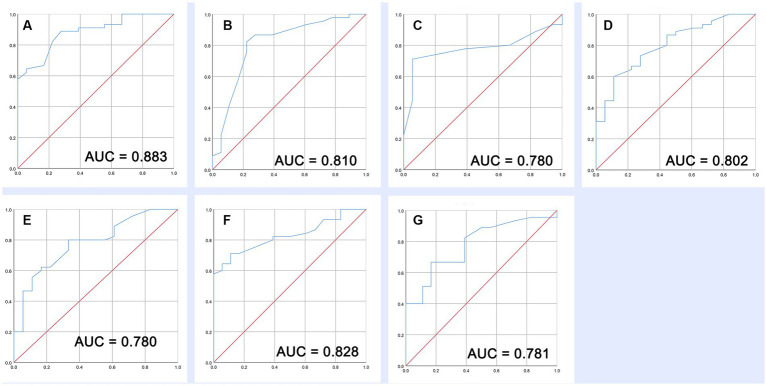
Prognostic value of increased fALFF and decreased FC. **(A–C)** ROC of three increased fALFF. **(D–G)** ROC of four decreased FC.

Based on the *Z*-values in ROI locations and FC changed regions, our prognosis prediction findings indicated that these increased or decreased fALFF and FC can effectively distinguish the long-term (6 months) outcomes in patients with sTBI at the early stage (AUC range from 0.780 to 0.883) (Figures 5A–G).

## Discussion

The most important innovation of this study is that we present a compensation mechanism at the acute phase of sTBI; that is, several parts of the brain regions can spontaneously activate and further lead to a decrease in functional connectivity (FC) to the whole brain, which may help to provide information for early diagnosis and prognostic prediction. Another innovation of this study is that we have carried out a gradual functional analysis framework of the sTBI patients’ residual brain activity; we use fALFF to identify the residual brain “activation” and use FC to describe its large-scale “connectome.” Significantly, several activations of brain areas reported in this study provide the early intervention targets for sTBI treatment.

To the best of our knowledge, both local activation and spatial connectivity form the prototype of various brain diseases (16, 18). There are two main methods for investigating the brain functional status in sTBI patients: one is to calculate the functional activity changes in the local ROI—*within* regions, and the other is to compare the functional interactions between regions, which can calculate the ROI-based FC to the whole brain over time—*between* regions (19). In this study, we found that fALFF values were mainly increased in the posterior cortex (e.g., occipital lobe, posterior cingulate gyrus, and parietal gyrus) of sTBI patients who present with good outcomes, indicating that these regions with high spontaneous brain functional activity are crucial for sTBI neurological function rehabilitation. The important clinical significance is that these results provide beneficial intervention targets for the early treatment of sTBI. It also reveals that these are essential compensatory brain regions that enable integration and coupling across the whole brain at the acute phase of sTBI (20). These findings consistently revealed that increased brain functional activity is associated with better cognitive performance in patients with chronic TBI (21). In addition, it has been reported that sTBI patients have lower values of local efficiency of their brain than healthy controls, implying altered brain functional organization (22). Our findings extend these findings by revealing an increased fALFF in the posterior cortex of sTBI patients who present with good outcomes, indicating that “from backward to forward” functional reorganization was involved in the good recovery of sTBI.

We further found that the injured brain can compensate itself by enhancing the fALFF signal of the local region, thereby reducing communication with the whole brain. Our ROI-based FC analysis results mainly focus on the brainstem region, indicating the contribution of subcortical regions to large-scale cortical region reorganization and the importance of FC between the cortex and subcortical regions in maintaining physiological function (23). For example, the functional pathway between the brainstem reticular ascending activation system and the cerebral cortex plays a critical role in wake-promoting at the acute phase of TBI (24). Accordingly, our results were not merely a consequence of the decrease in a few FC values, which was significantly associated with poorer balance of brain functional performance. Particularly, sTBI patients with poor outcomes displayed abnormally increased FC to massive brain regions; this reorganization phenomenon caused by injury reveals the importance of brain region coordination and cooperation in maintaining normal brain function (25).

It has been recently reported that the formation of brain functional architecture is an optimal trade-off between promoting communication efficiency and reducing wiring costs (26). At this point, these findings indicated that the synchronization of the whole brain is compromised in the acute phase of sTBI (27). Most interestingly, the dispersed functional hubs (increased in various FCs) could form a less efficient cortical network in the acute phase of sTBI. To the best of our knowledge, previous sTBI studies mainly emphasized a decrease in connectivity between the two ICNs (DMN and SN) (11, 28), and the residual FC could reappear in the chronic phase of sTBI (3 months after injury)(29). However, little is known about the functional compensation mechanisms at the acute phase of sTBI. Accordingly, our findings revealed that massive increased FC in the brain was associated with the severity of brain dysfunction at the acute phase of sTBI, which plays an important role in the poor prognosis in the long-term follow-up. To further investigate the clinical value of such abnormal brain functional patterns, we performed the predictive analysis of altered fALFF and FC on the long-term outcome of sTBI patients. Significantly, our findings suggested that sTBI patients with poor outcomes tend to have increased FC than those with good outcomes; it reveals that the less the compensatory effect of FC, the better the residual brain function of sTBI patients at the acute phase.

In the past, a lot of prognostic models have been developed for patients with sTBI, and the AUC of a model that separates the different outcomes in TBI is approximately 0.8. Although several complex models showed slightly better discrimination ability, most of them are broadly accepted in sTBI research rather than clinical practice (30). In this study, we developed severe high-quality indicators through fMRI scanning in the acute phase of sTBI. This objective evaluation method from the real clinical setting is comparable to complex prediction models of sTBI. Of which fALFF increase in the temporal lobe, occipital lobe, precuneus, and middle temporal gyrus shows the highest predictive ability (AUC = 0.883). These findings indicated that prognostic assessment based on brain functional data has great potential as it could reflect patient characteristics from multiple dimensions. As expected, we will try more schemes (functional and structural) in future research and compare the performance of different combinations.

## Limitations

This study has several limitations that should be highlighted. First, the sample size of this study was relatively small because of the complexity and difficulty of data collection during the acute phase of sTBI. Second, we excluded subjects with motion > 2 mm or 2 degrees of rotation in this study and its potential effect on signals analysis needs further clarification, and the macro-scale fMRI BOLD signals analysis cannot capture the compensation mechanism of sTBI at the neural circuits and molecular level. Third, although many potential confounding factors (cerebrospinal fluid, white matter fibers) have been adjusted, the data bias and the different injury forms or sites may affect the findings. In addition, whether these findings could be extended to other types of brain injury (e.g., cerebral hemorrhage or infarction) is not yet known.

## Conclusion

In summary, we present a compensatory mechanism that several regions of the brain can be spontaneously activated at the acute phase of sTBI in those who present with a good prognosis in the 6-month follow-up. The residual brain activities of sTBI could be dominated by the increased fALFF in the local region and decreased FC to the whole brain. Interestingly, such abnormal brain function pattern has a potential capability for predicting the 6-month outcome of patients with sTBI, of which fALFF increased in the temporal lobe, occipital lobe, precuneus, and middle temporal gyrus has the highest distinguishability.

## Data availability statement

The raw data supporting the conclusions of this article will be made available by the authors, without undue reservation.

## Ethics statement

The studies involving humans were approved by the Department of Emergency, the Second Affiliated Hospital of Nanchang University, Nanchang, Jiangxi, China. The studies were conducted in accordance with the local legislation and institutional requirements. Written informed consent for participation in this study was provided by the participants' legal guardians/next of kin.

## Author contributions

BL: Data curation, Formal analysis, Writing – original draft. WL: Data curation, Formal analysis, Writing – original draft. YG: Conceptualization, Writing – original draft, Methodology, Resources. YW: Writing – original draft, Formal analysis, Funding acquisition. LX: Methodology, Data curation, Formal analysis, Writing – review & editing. YY: Writing – original draft. SX: Writing – review & editing. ZT: Conceptualization, Investigation, Supervision, Writing – original draft. YM: Writing – review & editing. KW: Conceptualization, Data curation, Investigation, Methodology, Project administration, Writing – original draft, Writing – review & editing.

## Footnotes

^1^ https://www.fil.ion.ucl.ac.uk/spm/software/

^2^ http://rfmri.org/DPARSF

^3^ https://www.graphpad.com/scientific-software/prism/
