# Supplementary material for: Integrating fractional amplitude of low-frequency fluctuation and functional connectivity to investigate the mechanism and prognosis of severe traumatic brain injury
Source: Front Neurol. 2023 Dec 8;14:1266167. doi: 10.3389/fneur.2023.1266167 (PMC10748505; doi:10.3389/fneur.2023.1266167)
Supplement: Supplementary file 1 [file Data_Sheet_1.DOC]

Cluster 1

Number of voxels: 180

Peak MNI coordinate: 21 -63 24

Peak MNI coordinate region: // Right Cerebrum // Temporal Lobe // Sub-Gyral // White Matter // undefined // Precuneus_R (aal)

Peak intensity: 6.3858

# voxels structure

180 --TOTAL # VOXELS--

180 Right Cerebrum

154 White Matter

116 Sub-Gyral

91 Temporal Lobe

48 Occipital Lobe

47 Precuneus

47 Cuneus_R (aal)

36 Parietal Lobe

24 Precuneus_R (aal)

24 Occipital_Sup_R (aal)

21 Gray Matter

15 Calcarine_R (aal)

10 brodmann area 31

10 Occipital_Mid_R (aal)

9 Cuneus

6 brodmann area 18

5 Sub-lobar

5 brodmann area 7

5 Extra-Nuclear

3 Middle Temporal Gyrus

----------------------

Cluster 2

Number of voxels: 165

Peak MNI coordinate: 57 -39 18

Peak MNI coordinate region: // Right Cerebrum // Temporal Lobe // Superior Temporal Gyrus // undefined // undefined // Temporal_Sup_R (aal)

Peak intensity: 5.9741

# voxels structure

165 --TOTAL # VOXELS--

164 Right Cerebrum

125 Parietal Lobe

117 White Matter

74 SupraMarginal_R (aal)

63 Inferior Parietal Lobule

58 Supramarginal Gyrus

43 Temporal_Sup_R (aal)

41 Gray Matter

35 brodmann area 40

35 Temporal Lobe

32 Superior Temporal Gyrus

32 Angular_R (aal)

12 Parietal_Inf_R (aal)

7 Angular Gyrus

4 Insula

4 Sub-lobar

3 brodmann area 13

2 brodmann area 42

1 brodmann area 39

----------------------

Cluster 3

Number of voxels: 223

Peak MNI coordinate: 0 -78 36

Peak MNI coordinate region: // Inter-Hemispheric // undefined // Precuneus // undefined // undefined // Cuneus_L (aal)

Peak intensity: 5.8535

# voxels structure

223 --TOTAL # VOXELS--

177 Left Cerebrum

137 Parietal Lobe

136 Precuneus

96 White Matter

78 Gray Matter

53 Precuneus_L (aal)

45 Cuneus_L (aal)

44 brodmann area 7

36 Occipital Lobe

36 Occipital_Sup_L (aal)

32 Limbic Lobe

31 Parietal_Sup_L (aal)

29 Right Cerebrum

28 Cingulate Gyrus

21 brodmann area 31

18 Cuneus

16 Cingulum_Post_L (aal)

14 Precuneus_R (aal)

14 Inter-Hemispheric

11 brodmann area 19

11 Sub-Gyral

11 Superior Parietal Lobule

11 Cingulum_Post_R (aal)

7 Cingulum_Mid_L (aal)

4 Occipital_Mid_L (aal)

3 Posterior Cingulate

2 Cingulum_Mid_R (aal)

2 Cuneus_R (aal)

2 brodmann area 23

1 Temporal Lobe

>>
