# Supplementary material for: Integrating fractional amplitude of low-frequency fluctuation and functional connectivity to investigate the mechanism and prognosis of severe traumatic brain injury
Source: Front Neurol. 2023 Dec 8;14:1266167. doi: 10.3389/fneur.2023.1266167 (PMC10748505; doi:10.3389/fneur.2023.1266167)
Supplement: Supplementary file 2 [file Data_Sheet_2.DOC]

Cluster 1

Number of voxels: 271

Peak MNI coordinate: -12 -18 -48

Peak MNI coordinate region: // undefined // undefined // undefined // undefined // undefined // undefined

Peak intensity: -5.4878

# voxels structure

271 --TOTAL # VOXELS--

113 Pons

68 Right Brainstem

52 Left Brainstem

23 Limbic Lobe

20 Right Cerebrum

17 Uncus

15 Gray Matter

11 ParaHippocampal_R (aal)

7 Medulla

6 White Matter

6 brodmann area 28

6 Parahippocampa Gyrus

5 brodmann area 36

4 ParaHippocampal_L (aal)

3 Left Cerebrum

3 brodmann area 35

1 brodmann area 34

>>
