# Supplementary material for: Integrating fractional amplitude of low-frequency fluctuation and functional connectivity to investigate the mechanism and prognosis of severe traumatic brain injury
Source: Front Neurol. 2023 Dec 8;14:1266167. doi: 10.3389/fneur.2023.1266167 (PMC10748505; doi:10.3389/fneur.2023.1266167)
Supplement: Supplementary file 3 [file Data_Sheet_3.DOC]

Cluster 1

Number of voxels: 171

Peak MNI coordinate: 18 -12 -45

Peak MNI coordinate region: // undefined // undefined // undefined // undefined // undefined // undefined

Peak intensity: -3.6048

# voxels structure

171 --TOTAL # VOXELS--

61 Right Cerebrum

56 Limbic Lobe

49 Uncus

39 Gray Matter

34 ParaHippocampal_R (aal)

23 brodmann area 28

18 White Matter

8 Right Brainstem

8 Pons

7 Fusiform_R (aal)

6 Parahippocampa Gyrus

5 Temporal Lobe

4 Inferior Temporal Gyrus

4 brodmann area 34

4 brodmann area 20

2 Amygdala

2 brodmann area 35

2 brodmann area 36

2 brodmann area 38

2 Temporal_Inf_R (aal)

1 Amygdala_R (aal)

1 Sub-Gyral

1 Temporal_Pole_Sup_R (aal)

1 Superior Temporal Gyrus

----------------------

Cluster 2

Number of voxels: 269

Peak MNI coordinate: 48 -3 9

Peak MNI coordinate region: // Right Cerebrum // Frontal Lobe // Precentral Gyrus // Gray Matter // brodmann area 6 // Rolandic_Oper_R (aal)

Peak intensity: -3.0277

# voxels structure

269 --TOTAL # VOXELS--

268 Right Cerebrum

133 White Matter

111 Gray Matter

90 Parietal Lobe

88 Frontal Lobe

83 Rolandic_Oper_R (aal)

74 Precentral Gyrus

64 Postcentral Gyrus

63 Sub-lobar

55 Insula

53 Postcentral_R (aal)

47 SupraMarginal_R (aal)

29 brodmann area 6

27 Temporal Lobe

20 brodmann area 13

20 Inferior Parietal Lobule

20 Insula_R (aal)

19 Sub-Gyral

19 Temporal_Sup_R (aal)

18 Superior Temporal Gyrus

16 brodmann area 43

13 brodmann area 4

12 brodmann area 40

9 Extra-Nuclear

8 brodmann area 41

7 Transverse Temporal Gyrus

4 brodmann area 3

3 brodmann area 1

3 brodmann area 44

3 Heschl_R (aal)

3 Frontal_Inf_Oper_R (aal)

2 Inferior Frontal Gyrus

2 Temporal_Mid_R (aal)

2 Precentral_R (aal)

1 brodmann area 2
