# Supplementary material for: Integrating fractional amplitude of low-frequency fluctuation and functional connectivity to investigate the mechanism and prognosis of severe traumatic brain injury
Source: Front Neurol. 2023 Dec 8;14:1266167. doi: 10.3389/fneur.2023.1266167 (PMC10748505; doi:10.3389/fneur.2023.1266167)
Supplement: Supplementary file 4 [file Data_Sheet_4.DOC]

Cluster 1

Number of voxels: 343

Peak MNI coordinate: -9 -18 -48

Peak MNI coordinate region: // undefined // undefined // undefined // undefined // undefined // undefined

Peak intensity: -4.5719

# voxels structure

343 --TOTAL # VOXELS--

56 Pons

51 Right Cerebrum

50 Limbic Lobe

47 Uncus

31 Gray Matter

30 Right Brainstem

28 Left Brainstem

24 White Matter

22 Fusiform_R (aal)

12 brodmann area 36

11 ParaHippocampal_R (aal)

9 Inferior Temporal Gyrus

8 Temporal Lobe

7 brodmann area 28

7 Left Cerebrum

7 brodmann area 20

4 Cerebelum_10_L (aal)

4 Fusiform_L (aal)

4 Temporal_Inf_R (aal)

4 Temporal_Pole_Mid_R (aal)

4 ParaHippocampal_L (aal)

3 brodmann area 38

2 Medulla

2 Parahippocampa Gyrus

2 brodmann area 35

1 Cerebellar Tonsil

1 Cerebellum Posterior Lobe

1 Temporal_Pole_Mid_L (aal)

1 Left Cerebellum

>>
